# Modulation of the sympathetic nervous system by renal denervation prevents reduction of aortic distensibility in atherosclerosis prone ApoE-deficient rats

**DOI:** 10.1186/s12967-016-0914-9

**Published:** 2016-06-08

**Authors:** Mathias Hohl, Dominik Linz, Peter Fries, Andreas Müller, Jonas Stroeder, Daniel Urban, Thimoteus Speer, Jürgen Geisel, Björn Hummel, Ulrich Laufs, Stephan H. Schirmer, Michael Böhm, Felix Mahfoud

**Affiliations:** Klinik für Innere Medizin III, Universität des Saarlandes, 66421 Homburg/Saar, Germany; Klinik für Diagnostische und Interventionelle Radiologie, Universität des Saarlandes, Homburg/Saar, Germany; Klinik für Innere Medizin IV, Universität des Saarlandes, Homburg/Saar, Germany; Zentrallabor, Klinische Chemie und Laboratorium Medizin, Universität des Saarlandes, Homburg/Saar, Germany; Institut für Klinische Hämostaseologie und Transfusionsmedizin, Universität des Saarlandes, Homburg/Saar, Germany

**Keywords:** ApoE-deficient rats, Hypercholesterolemia, Aortic distensibility, Renal sympathetic denervation

## Abstract

**Background:**

Apolipoprotein E-deficient (ApoE^−/−^) rodents spontaneously develop severe hypercholesterolemia and increased aortic stiffness, both accepted risk factors for cardiovascular morbidity and mortality in humans. In patients with resistant hypertension renal denervation (RDN) may improve arterial stiffness, however the underlying mechanisms are incompletely understood. This study investigates the impact of RDN on aortic compliance in a novel atherosclerosis prone ApoE^−/−^-rat model.

**Methods:**

Normotensive, 8 weeks old ApoE^−/−^ and Sprague–Dawley (SD) rats were subjected to bilateral surgical RDN (n = 6 per group) or sham operation (n = 5 per group) and fed with normal chow for 8 weeks. Compliance of the ascending aorta was assessed by magnetic resonance imaging. Vasomotor function was measured by aortic ring tension recordings. Aortic collagen content was quantified histologically and plasma aldosterone levels were measured by enzyme-linked immunosorbent assay (ELISA).

**Results:**

After 8 weeks, ApoE^−/−^-sham demonstrated a 58 % decrease in aortic distensibility when compared with SD-sham (0.0051 ± 0.0011 vs. 0.0126 ± 0.0023 1/mmHg; p = 0.02). This was accompanied by an impaired endothelium-dependent relaxation of aortic rings and an increase in aortic medial fibrosis (17.87 ± 1.4 vs. 12.27 ± 1.1 %; p = 0.006). In ApoE^−/−^-rats, RDN prevented the reduction of aortic distensibility (0.0128 ± 0.002 vs. 0.0051 ± 0.0011 1/mmHg; p = 0.01), attenuated endothelial dysfunction, and decreased aortic medial collagen content (12.71 ± 1.3 vs. 17.87 ± 1.4 %; p = 0.01) as well as plasma aldosterone levels (136.33 ± 6.6 vs. 75.52 ± 8.4 pg/ml; p = 0.0003). Cardiac function and metabolic parameters such as hypercholesterolemia were not influenced by RDN.

**Conclusion:**

ApoE^−/−^-rats spontaneously develop impaired vascular compliance. RDN improves aortic distensibility and attenuated endothelial dysfunction in ApoE^−/−^-rats. This was associated with a reduction in aortic fibrosis formation, and plasma aldosterone levels.

**Electronic supplementary material:**

The online version of this article (doi:10.1186/s12967-016-0914-9) contains supplementary material, which is available to authorized users.

## Background

The ability of the arterial system to adapt to changes in mechanical demands depends on its vascular compliance. Compliance describes the amount of volume change of blood vessels following a given (blood) pressure change [1]. Hyperlipidemia can induce remodeling processes of the arterial wall, thereby impairing vascular compliance [2–4]. The underlying molecular mechanisms contributing to impaired vascular compliance are multifactorial and may involve increased sympathetic nerve activity leading to renin-angiotensin-aldosterone-system (RAAS) activation, enhanced vascular oxidative stress, and overexpression of inflammatory molecules [4–8].

Renal denervation (RDN) has been shown to reduce blood pressure (BP) in animal models and in certain patients with uncontrolled hypertension [9–12]. Recent studies have demonstrated that RDN may also exhibit additional systemic effects in parallel to BP lowering [13, 14], including improvement in arterial stiffness [15]. In obese, spontaneously hypertensive rats (SHR-ob), RDN inhibited progressive BP increase in SHR-ob and suppressed progression of kidney injury, cardiac fibrosis, and left ventricular dysfunction [16]. In goats with persistent atrial fibrillation (AF), RDN reduced atrial sympathetic nerve sprouting, structural alterations, and AF complexity [17]. In normotensive ApoE-deficient mice, fed with Western-type diet, RDN attenuated atherosclerotic lesion formation at the aortic root without affecting hyperlipidemia [18].

The effect of RDN on the development of aortic remodeling and impairment of vascular compliance under hyperlipidemic conditions yet at a preatherosclerotic stage has not yet been investigated systematically. To assess the role of the sympathetic nervous system and its modulation by RDN, we used a novel, normotensive ApoE-deficient rat model of hypercholesterolemia [19] with a hitherto uncharacterized cardiovascular phenotype.

## Methods

All chemicals used were purchased from Carl Roth GmBH + Co.KG (Karlsruhe, Germany) unless mentioned otherwise.

### Animals

The animal experiments were conducted in accordance with “The guide for the Care and Use of laboratory Animals” published by the US National Institutes of Health (NIH Publication No. 85-23, revised 1996) and approved by the local animal ethics committee (Landesamt für Verbraucherschutz, Amtstierärztlicher Dienst, Lebensmittelüberwachung Zentralstelle, Saarbrücken, Germany. Nr.: 38/2012). Male ApoE-deficient rats (#TGRA3710HM4-1EA; ApoE^−/−^, n = 11), and their littermate controls Sprague–Dawley (SD, n = 11), were purchased from Sigma-Aldrich-SAGE Labs (Boyertown, USA) at an age of 7 weeks. The animals were housed individually in standard cages and received standard chow diet (Ssniff^®^ standard diet #V1536, Ssniff^®^ Spezialitäten GmbH, Soest, Germany) and tap water ad libitum. At an age of 8 weeks the animals were randomized to either RDN or sham operation and sacrificed at an age of 16 weeks. Blood pressure (BP) and heart rate (HR) were measured non-invasively by a computerized tail-cuff system (BP-2000 Visitech Systems, USA).

It is known from ApoE-deficient mice that usage of high-fat, high-cholesterol Western-type diet accelerated the progression of atherosclerosis [18, 20]. In ApoE^−/−^-rats, we observed that administration of a Western-type diet containing 0.3 % cholesterol resulted in increased mortality after 2–3 weeks, most likely due to severe fatty degeneration of the liver (Additional file 1: Figure S1). Therefore, we resigned from feeding ApoE^−/−^-rats with Western-type diet and focused on preatherosclerotic aortic remodeling in hyperlipidemic ApoE^−/−^-rats at an age of 16 weeks fed with standard chow [19].

### Renal denervation and sham operation

RDN in rats was performed as described previously [16]. Six ApoE^−/−^ and six SD rats (ApoE^−/−^-RDN and SD-RDN, respectively) underwent RDN at the age of 8 weeks. In brief, rats were anesthetized with 1.5–2.5 % isoflurane (AbbVie #B506, Ludwigshafen, Germany) and both kidneys were surgically denervated by cutting all visible nerves in the area of the renal hilus and by stripping approximately 2–4 mm of the adventitia from the renal artery and moistening with a 20 % phenol/ethanol solution. Another five ApoE^−/−^ and five SD rats were submitted to a surgical sham procedure with kidney exposition but without RDN, serving as sham controls (ApoE^−/−^-sham and SD-sham, respectively). At an age of 16 weeks, rats were sacrificed and reductions in renal tissue norepinephrine (NE) concentrations were determined by high-performance liquid chromatography to assess the effectiveness of RDN.

### Magnetic resonance imaging

All MRI experiments were performed using a 9.4 Tesla horizontal bore animal scanner (Bruker BioSpin 94/20, Ettlingen, Germany) with a 2 × 2 phased-array surface coil. All animals were subjected to general anesthesia using a mixture of isoflurane and oxygene (2 %/98 %) applied by a dedicated nose mask at a flow rate of 1.5 l/min. A retrospectively self-gated black blood sequence (IntraGate FLASH, TR 8.9 ms, TE 2.1 ms, flip angle 10°, field of view 4.5 × 4.5 cm, matrix 384 × 384, slice thickness 1 mm, pixel size 117 × 117 μm^2^) was acquired perpendicular to the ascending aorta at the level of the right pulmonary artery. 25 cine frames were reconstructed from the acquired raw data in order to display one cardiac cycle. In addition, retrospectively self-gated bright blood cine sequence were acquired in short axis orientation covering the left ventricle from base to apex with reconstruction of 20 cine frames for every dedicated slice position (IntraGate FLASH, TR 5.6 ms, TE 1.5 ms, flip angle 12°, field of view 4.0 × 4.0 cm, matrix 256 × 256, slice thickness 1 mm, pixel size 156 × 156 μm^2^). For further quantitative analyses, the image data were transferred to an external workstation and evaluated using image evaluation software (OsiriX^®^, Pixmeo, Bernex, Switzerland).

For the assessment of aortic distensibility the cross sectional vessel area of the ascending aorta was measured by manual contouring at the blood/vessel wall interface at end-diastole (ED) and end-systole (ES).

Aortic distensibility (AD) was calculated with

$${\text{AD}}\; = \;\left( {{\text{Area}}\;({\text{ES}})\; - \;{\text{Area }}\left( {\text{ED}} \right)/({\text{Area }}\left( {\text{ED}} \right) \times {\text{pulse pressure }}\left( {\text{PP}} \right)} \right),$$

Left ventricular end diastolic volume (LVEDV) and the left ventricular end systolic volume (LVESV) were evaluated by manual contouring of the blood/subendocardial myocardium.

Stroke volume (SV) and ejection fraction (EF) were calculated with:

$${\text{SV}} = {\text{LVEDV}} {-} {\text{LVESV}}$$

$${\text{EF}} = {\text{SV}}/{\text{LVEDV}}$$

### Histology

#### Aortic medial collagen content

Thoracic aorta was sectioned (10 μm) on a Leica cryostat at −25 °C (Kryostat Leica CM 1900-V5.0, Leica Microsystems, Nussloch, Germany). Sections were stained with Picro-Sirius Red (Morphisto^®^, Frankfurt, Germany) to visualize collagen fibers. The percentage of the media consisting of interstitial collagen was calculated as the ratio of Picro-Sirius-Red positively stained area over total aortic medial-tissue area. For the analysis Nicon Instruments Software (NIS)-Elements (BR 3.2, Nikon instruments, USA) was used.

#### Ficoll-isolation of mononuclear cells

Peripheral blood mononuclear cells (MNCs) were isolated by the use of a Ficoll density gradient within 30 min after collecting peripheral arterial blood from all animals. Whole citrate blood was mixed 1:1 (v/v) with a solution of phosphate-buffered saline (PBS) and layered onto 0.25 Vol. of Ficoll (Biochrom GmbH, Berlin, Germany). After centrifugation at 800*g* for 20 min at room temperature, the layer of mononuclear cells was collected and stored immediately for RNA isolation.

#### Gene expression

Reverse Transcription: RNA was prepared from rat aortic-tissue and mononuclear cells using peqGold TriFast (PeqLab, Erlangen, Germany) extraction reagent per manufacturer’s protocol. For cDNA preparation 2 μg of RNA were digested with DNAse (Peqlab) than reversely transcribed using the HighCap cDNA RT Kit (Applied Biosystems, Darmstadt, Germany) according to the manufacturer´s protocol. TaqMan PCR was conducted in a StepOne plus thermocycler (Applied Biosystems) using TaqMan GenEx Mastermix (Applied Biosystems, #4369016). Signals were normalized to corresponding glyceraldehyde-3-phosphate dehydrogenase (GAPDH) controls. No template controls were used to monitor for contaminating amplifications. The ΔCt was used for statistical analysis and 2^−ΔΔCt^ for data presentation. Probes used to amplify the transcripts were as follows (Applied Biosystems): TNFα (Rn01525859_g1); IL1β (Rn00580432_m1); IL6 (Rn01410330_m1); GAPDH (Rn99999916_s1), ICAM1 (Rn00564227_m1), VCAM1 (Rn00563627_m1); eNOS (Rn02132634_s1).

### Enzyme linked immunosorbent assays (ELISA)

Plasma concentrations of aldosterone, angiotensin II, IL6 and IL1β were determined by ELISA, using Enzo Aldosterone EIA Kit (ADI-900-173; Lausen, Switzerland), Enzo Angiotensin II ELISA Kit (ADI-900-204; Lausen, Switzerland), Rat IL6 ELISA Kit (R&D systems, Abington, UK) and Cloud IL1β Immunoassay (Cloud-Clone Corp. Houston, TX, USA) following the manufacturer’s protocol. For analysis of renal renin and angiotensin II tissue concentrations, 10 mg kidney of each rat was homogenized in 600 µl PBS, containing 1 % Triton-X and complete protease inhibitors (Roche Diagnostics GmbH, Mannheim, Germany), followed by centrifugation at 18000*g* for 5 min. Angiotensin II and renin levels were measured from the supernatant by ELISA using Elabscience ELISA Kit (WuHan, Peoples Republic of China) following the manufacturer’s protocol.

### Determination of renal norepinephrine content

Kidney tissue from rat was minced thoroughly in TRIS-EDTA buffer (in mmol/l: 5.0 Tris(hydroxymethyl)aminomethane, 2.0 Na-EDTA, pH 7.4). After centrifugation, supernatant was used to analyze NE content by high pressure liquid chromatography (HPLC) using Chromosystems catecholamine Kit for HPLC (Chromsystems Instruments & Chemicals GmbH, Munich, Germany) following the manufacturer’s protocol.

### Aortic ring preparation and tension recording

Aortic ring tension was measured as described previously [20]. After excision, the thoracic aorta was immersed in Tyrode’s solution containing (in mmol/l) 118.0 NaCl, 2.5 CaCl_2_, 4.73 KCl, 1.2 MgCl_2_, 1.2 KH_2_PO_4_, 25.0 NaHCO_3_, 0.026 Na-EDTA, and 5.5 d-(+)-glucose, pH 7.4. Adventitial tissue was carefully removed. Three-millimeter aortic rings (n = 4 rings per animal with n = 5 animals per group) were mounted in organ bath chambers filled with the Tyrode’s solution (37 °C; aerated with 95 % O_2_ and 5 % CO_2_) and were attached to a force transducer recording isometric tension (Model 750TOBS, Danish Myo Technology (DMT), Aarhus, Denmark). Aortic rings were stretched to a resting tension of 10 mN, which was maintained throughout the experiment. Pharmacologically induced contraction of aortic rings was performed with phenylephrine (5 μmol/l; Sigma-Aldrich, Steinheim, Germany). After a plateau was reached, increasing concentrations of carbachol (carbamylcholine chloride; 1 nmol/l–100 μmol/l; Sigma-Aldrich) were added to obtain cumulative concentration—response curves to study endothelium-dependent relaxation. The relaxing effect of carbachol was abolished by adding *N*-nitro-l-arginine methyl ester (1 μmol/l; Sigma-Aldrich). To study endothelium-independent relaxation after a washout period, increasing concentrations of glyceryl trinitrate (100 nmol/l–10 µmol/l; Sigma-Aldrich) were added after a single dose of phenylephrine (5 µmol/l).

Aortic rings without any response to relaxation with carbachol or glyceryl trinitrate (<10 % relaxation) were excluded from statistical analysis. Relaxation response was expressed as percentage of phenylephrine-induced pre-contraction. The concentration of drugs used to achieve 50 % of the maximal response (logEC_50_) was determined by using nonlinear regression analysis (GraphPad Prism 6, San Diego, CA, USA).

### Statistics

Data are presented as mean ± SEM and differences were tested for significance using an unpaired Student’s *t* test, one-way-ANOVA with Tukey’s multiple comparisons test or two-way-ANOVA with Bonferroni multiple comparisons test when appropriate. A p value of less than 0.05 was considered statistically significant. Statistical analysis was performed using GraphPad Prism 6.

## Results

### Renal norepinephrine content, cardiac function, and metabolic parameters

**Fig. 1:**
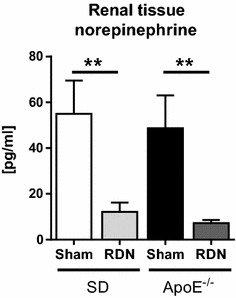
Effect of RDN on renal norepinephrine content. Kidney tissue norepinephrine (NE) content of ApoE^−/−^ rats and SD controls, 8 weeks after RDN- (n = 6 per group) or sham-operation (n = 5 per group). Values are mean ± SEM. **p > 0.01

Eight weeks after RDN-procedure, renal NE concentrations were significantly lower in SD-RDN and ApoE^−/−^-RDN, when compared to their respective sham-operated controls (Fig. 1), confirming successful renal nerve ablation. Table 1 depicts parameters of cardiac function and serum chemistry eight weeks after RDN- or sham intervention. Body weight, heart weight, systolic and diastolic blood pressure, heart rate, stroke volume, ejection fraction, left ventricular systolic, and diastolic volume did not differ between ApoE^−/−^-sham and SD-sham, demonstrating that at an age of 16 weeks ApoE^−/−^-rats remain normotensive. Blood pressure did not differ between ApoE^−/−^ and SD rats at the age of 8 weeks. Total cholesterol, low-density lipoprotein (LDL), and triglycerides were significantly higher in ApoE^−/−^-rats, while high-density lipoprotein (HDL) was significantly lower. RDN had no influence on myocardial function or metabolic parameters.

**Table 1:** Body weight, blood pressure and metabolic parameters 8 weeks after RDN

	SD-sham n = 5	SD-RDN n = 6	ApoE^−/−^-sham n = 5	ApoE^−/−^-RDN n = 6
Body weight (g)	456.2 ± 25.64	474.14 ± 11.26	446.75 ± 3.68	456.2 ± 10
Heart weigth (g)	1.41 ± 0.04	1.45 ± 0.05	1.51 ± 0.06	1.45 ± 0.04
Heart rate (bpm)	360 ± 9	370 ± 10	341 ± 13	359 ± 7
Blood pressure (mmHg)				
Systolic	144 ± 5	137 ± 4	148 ± 12	136 ± 7
Diastolic	116 ± 4	109 ± 10	121 ± 11	99 ± 8
Stroke volume (µl)	210 ± 10	219 ± 5	203 ± 10	224 ± 9
Ejection fraction (%)	61 ± 3	61 ± 1	59 ± 2	60 ± 2
LVEDV (µl)	342 ± 11	357 ± 11	344 ± 8	372 ± 13
LVESV (µl)	131 ± 12	137 ± 8	140 ± 11	148 ± 10
Serum chemistry				
GPT (U/l)	79.64 ± 7.53	70.5 ± 5.91*	109.66 ± 14.96	104.43 ± 10.26
GOT (U/l)	178.36 ± 24.74	169.64 ± 13.83	222.22 ± 33.31	217.28 ± 32.1
Creatinine (mg/dl)	0.306 ± 0.02	0.33 ± 0.01	0.33 ± 0.02	0.29 ± 0.03
Glucose (mg/dl)	235.2 ± 8.24	268.71 ± 17.18	328.25 ± 28.51	289.33 ± 25.65
Cholesterol (mg/dl)	48.2 ± 3.28^§^	45.71 ± 3.21^#^	131.5 ± 17.54	152.5 ± 13.63
Triglycerides (mg/dl)	30.6 ± 7.04^§^	30.57 ± 8.05^#^	152.2 ± 27.41	152.5 ± 18.44
LDL (mg/dl)	10.96 ± 1.15^§^	10.5 ± 0.94^#^	52.3 ± 6.62	65.28 ± 9.61
HDL (mg/dl)	37.58 ± 1.36^§^	34.93 ± 3.36^#^	11.7 ± 1.67	15.78 ± 1.81

Values are mean ± SEM

*LVEDV* left ventricular end-diastolic volume, *LVESV* left ventricular end-systolic volume, *GPT* glutamat-pyruvat-transaminase, *GOT* glutamat-oxalacetat-transaminase, *HDL* high-density lipoprotein, *LDL* low-density lipoprotein

* p < 0.05 SD-RDN vs. ApoE^−\−^-RDN

^§^p < 0.01 SD-sham vs. ApoE^−\−^-sham

^#^p < 0.01 SD-RDN vs. ApoE^−\−^-RDN (one-way-Anova followed by Tukey´s multiple comparisons test)

### RDN improved aortic distensibility and attenuated progression of endothelial-dependent dysfunction

At an age of 16 weeks, ApoE^−/−^-sham rats exhibited a significantly reduced aortic distensibility compared with age-matched SD-rats (0.0051 ± 0.0011 vs. 0.0126 ± 0.0023 1/mmHg; p = 0.02), as measured by MRI with high-resolution cine sequences acquired at 9.4 Tesla. ApoE^−/−^-rats also demonstrated an age-dependent reduction of aortic distensibility, comparing baseline values at an age of 8 weeks (0.0138 ± 0.0039 1/mmHg) and final measurements at an age of 16 weeks (0.0051 ± 0.0011 1/mmHg; p = 0.07). Lack of statistical significance might by due to the limited number of animals.

**Fig. 2:**
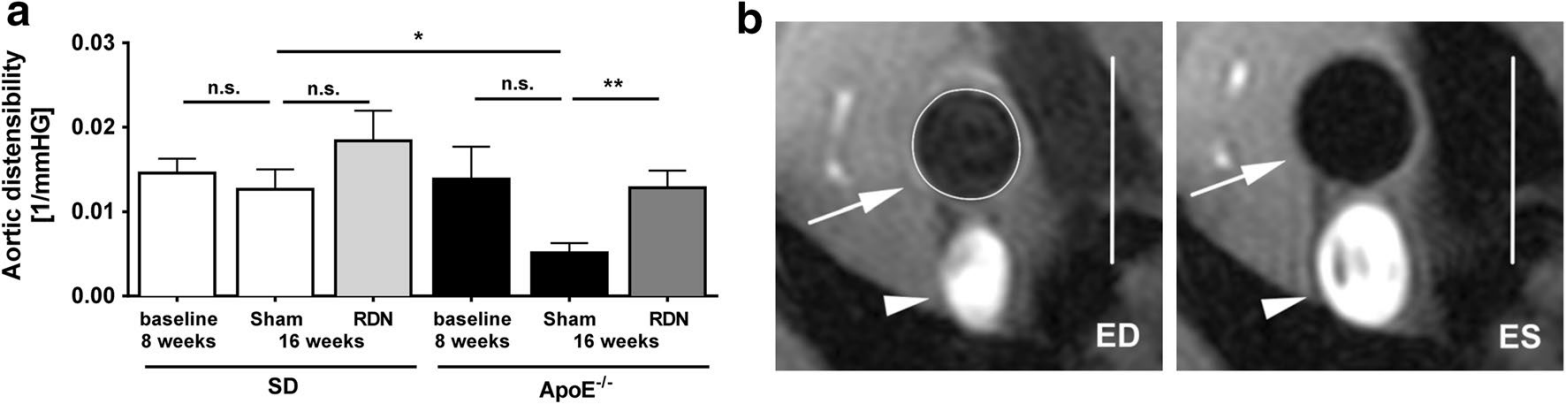
Effect of RDN on aortic distensibility and vasorelaxation. **a** Quantification of aortic distensibility at baseline (n = 6 per group), and 8 weeks after RDN procedure (n = 6 per group) or sham operation (n = 5 per group). Data are based on in vivo measurements of cross section vessel areas. **b** Representative example of high-resolution *black* blood cine MRI perpendicular to the ascending aorta. The *bar* on the *right side* indicates 5 mm. The *circle* depicts the cross sectional vessel area of the ascending aorta (*arrow*). The *arrowhead points* to the superior caval vein. *ED* end diastole, *ES* end systole. Values are mean ± SEM. SD baseline vs. SD-sham, p = 0.51 (n.s.); SD-sham vs. SD-RDN, p = 0.23 (n.s.); ApoE^−/−^ baseline vs. ApoE^−/−^-sham, p = 0.07 (n.s.); *p < 0.05, **p < 0.01

**Fig. 3:**
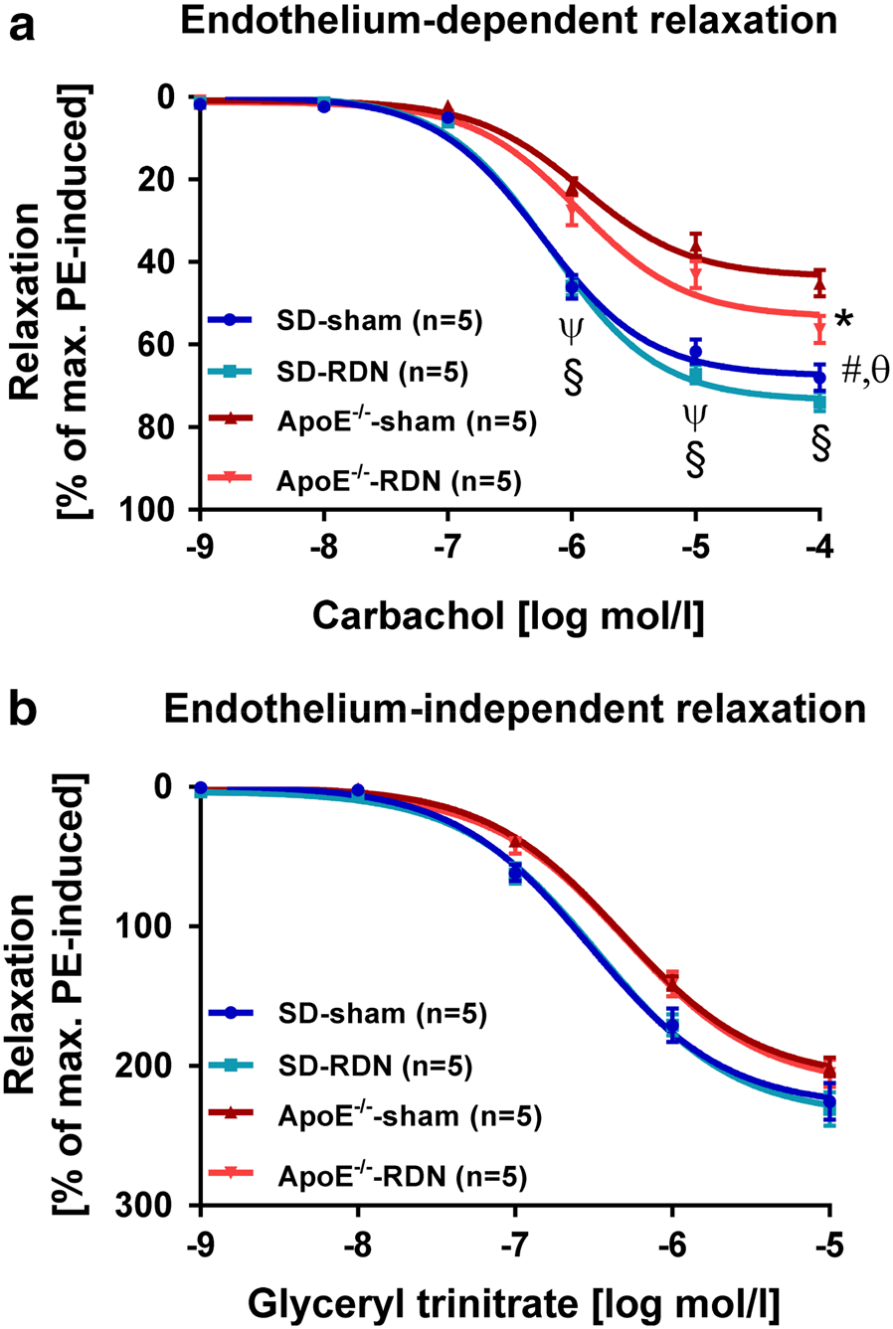
Effect of RDN on endothelial function. **a** Endothelium-dependent vasorelaxation of aortic rings in response to carbachol and **b** endothelium-independent relaxation in response to glyceryl trinitrate in 16 weeks old SD-sham, SD-RDN, ApoE^−/−^-sham and ApoE^−/−^-RDN rats. Values are mean ± SEM. ^§^p < 0.001 SD-RDN vs. ApoE^−\−^-RDN and ApoE^−\−^-sham; ^ψ^p < 0.001 SD-sham vs. ApoE^−\−^-RDN;#p < 0.01 SD-sham vs. ApoE^−\−^-RDN; ^θ^p < 0.001 SD-sham vs. ApoE^−\−^-sham; *p < 0.01 ApoE^−\−^-RDN vs. ApoE^−\−^-sham (two-way-Anova followed by Bonferroni multiple comparison test)

In ApoE^−/−^-RDN rats, reduction of aortic distensibility was prevented (0.0128 ± 0.002 (1/mmHg; p = 0.01 vs. ApoE-sham) and equals values of SD-sham rats (Fig. 2a, b). Endothelial relaxation of aortic rings was tested in organ bath experiments at an age of 16 weeks. Efficacy of endothelium-dependent vascular response to increasing concentrations of carbachol was significantly impaired in ApoE^−/−^-sham compared with SD-sham. In ApoE^−/−^ receiving RDN, vasorelaxation improved when compared with ApoE^−/−^-sham animals (Fig. 3a). Calculation of the maximal response (*E*_max_) and half-maximal dose (logEC_50_) to Carbachol also demonstrated a reduced response of the endothelium in ApoE^−/−^-sham compared to SD-sham. However, RDN-treatment of ApoE^−/−^ failed to reach significant improvement of endothelial relaxation using linear regression calculation (Table 2). Endothelium-independent relaxation induced by glyceryl trinitrate did not significantly differ between the groups (Fig. 3b). *E*_max_ and logEC_50_ of glyceryl trinitrate did also not differ among the groups (Table 2).

**Table 2:** Maximal relaxation (*E*_max_) and half-maximal dose (logEC_50_) in response to carbachol and glyceryl trinitrate in 16 weeks old rats

	SD-sham	SD-RDN	ApoE^−/−^-sham	ApoE^−/−^-RDN
Number of rats	5	5	5	5
Response to carbachol				
*E*_max_, (%)	67 ± 6.0	73 ± 3.3	43 ± 4.8^§^	53 ± 4.9
logEC_50_ (log M)	−6.22 ± 0.06	−6.17 ± 0.03	−5.81 ± 0.15	−5.88 ± 0.12
Response to glyceryl trinitrate				
*E*_max_, (%)	232 ± 24	237 ± 6	209 ± 13	217 ± 16
logEC_50_ (log M)	−6.5 ± 0.10	−6.47 ± 0.08	−6.32 ± 0.06	−6.29 ± 0.10

Values are mean ± SEM. p values were calculated using one-way-Anova followed by Tukey´s multiple comparisons test

^§^p = 0.01 SD-sham vs. ApoE^−\−^-sham

Additionally, we analyzed mRNA expression pattern of endothelial nitric oxide synthase (eNOS) in aortic tissue of ApoE^−\−^ and SD rats. Levels of mRNA were not differentially regulated between the groups and RDN had no effect on eNOS gene expression (Additional file 1: Table S1).

### Effect of RDN on RAAS activation and inflammatory response

**Fig. 4:**
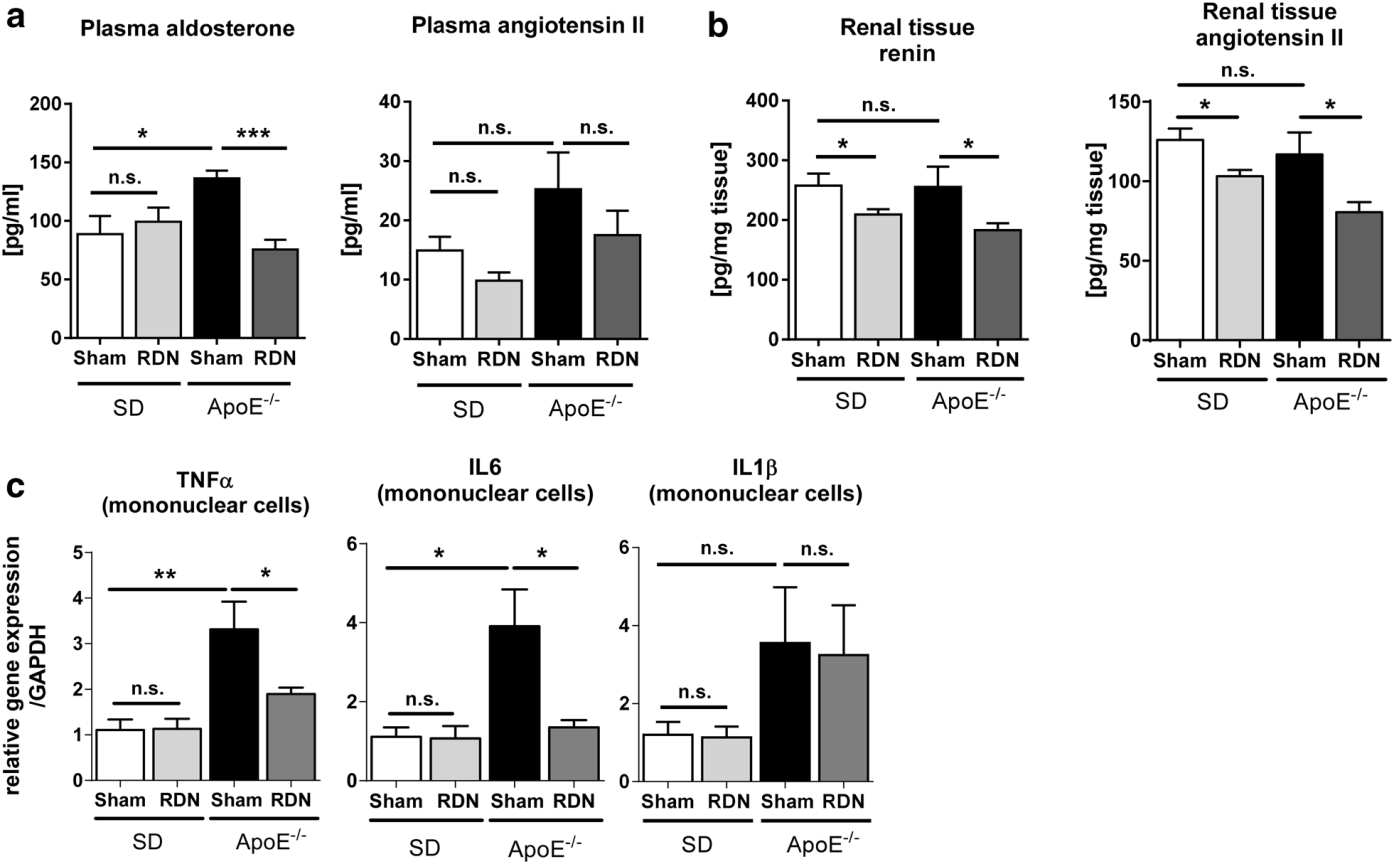
Effect of renal denervation (RDN) on the renin-angiotensin-aldosterone system (RAAS) and on expression of inflammatory cytokines in isolated mononuclear cells. **a** Quantification of plasma aldosterone and angiotensin II. **b** Evaluation of renal tissue level of renin and angiotensin II **c** quantification of mRNA expression levels in mononuclear cells (MNCs) of IL1β, IL6 and TNFα in SD-sham (n = 5), SD-RDN (n = 6), ApoE^−\−^-sham (n = 5), ApoE^−\−^-RDN (n = 6). Values are mean ± SEM. Plasma aldosterone: SD-sham vs. SD-RDN, p = 0.60 (n.s.); Plasma angiotensin II: SD-sham vs. ApoE^−/−^-sham, p = 0.15 (n.s.); ApoE-sham vs. ApoE^−/−^-RDN, p = 0.07 (n.s.); SD-sham vs. SD-RDN, p = 0.08 (n.s.). Tissue renin SD-sham vs. ApoE^−/−^-sham, p = 0.93 (n.s.). Tissue angiotensin II SD-sham vs. ApoE^−/−^-sham, p = 0.57 (n.s.). TNFα SD-sham vs. SD-RDN, p = 0.78 (n.s.); IL6 SD-sham vs. SD-RDN, p = 0.91 (n.s.); IL1β SD-sham vs. SD-RDN, p = 0.86 (n.s.); ApoE^−\−^-sham vs. ApoE^−\−^-RDN, p = 0.87 (n.s.); SD-sham vs. ApoE^−\−^-sham, p = 0.14 (n.s.); *p < 0.05, **p < 0.01; ***p < 0.001

Plasma aldosterone concentration was significantly increased in ApoE^−/−^-sham compared with SD-sham (136.33 ± 6.6 vs. 88.83 ± 15.4 pg/ml; p = 0.02). Enhanced aldosterone release was inhibited following RDN in ApoE^−/−^ (75.52 ± 8.4 pg/ml; p = 0.0003 vs. ApoE-sham), but not in SD-RDN (99.17 ± 12.2 pg/ml; p = 0.60 vs. SD-sham) (Fig. 4a). AngII plasma level was not changed in ApoE^−/−^-sham compared with SD-sham (25.26 ± 6.2 vs. 14.92 ± 2.3 pg/ml; p = 0.15) and RDN-treatment did not significantly reduce AngII plasma level neither in ApoE^−/−^-RDN (17.51 ± 4.1 pg/ml; p = 0.07 vs. ApoE-sham) nor in SD-RDN (9.82 ± 1.4 pg/ml; p = 0.08 vs. SD-sham). Renal tissue concentration of renin and AngII did not differ between sham-operated rats, however RDN resulted in a significant reduction of renin and AngII-tissue level in ApoE^−/−^-RDN and SD-RDN (Fig. 4b).

In circulating mononuclear cells, gene expression of TNFα and IL6 was significantly increased in ApoE^−/−^-sham compared with SD-sham, while IL1β was unchanged. RDN significantly inhibited transcriptional enhancement of TNFα and IL6 in ApoE^−/−^, but had no effect on IL1β mRNA-levels (Fig. 4c). Plasma levels of IL6 and IL1β were significantly higher in ApoE^−/−^-sham compared to SD-sham (IL6: 157.06 ± 6.54 vs. 123.78 ± 4.39 pg/ml, p < 0.01; IL1β: 177.41 ± 8.77 vs. 65.75 ± 15.32 pg/ml, p < 0.01) demonstrating increased inflammation in ApoE^−/−^ rats. Modulation of the sympathetic nervous system by RDN had no effect on IL6 or IL1β plasma concentration, neither in SD-RDN nor in ApoE^−/−^-RDN (Additional file 1: Table S1).

In aortic tissue of ApoE^−/−^-sham, IL1β mRNA-levels were elevated in comparison to SD-sham (relative gene expression/GAPDH: 2.55 ± 0.3 vs. 1.15 ± 0.2; p = 0.02), while transcription of TNF and pro-atherosclerotic intercellular adhesion molecule-1 (ICAM-1) and vascular cell adhesion molecule 1 (VCAM-1) were unchanged. RDN could neither attenuate inflammatory nor pro-atherosclerotic gene expression (Additional file 1: Table S1).

### Morphological analysis of the thoracic aortic wall

**Fig. 5:**
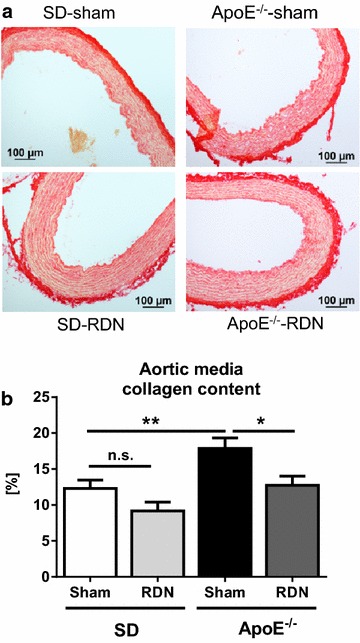
Effect of RDN on aortic medial fibrosis content. **a** Representative *Sirius-red* staining for determination of interstitial collagen content of the aortic media. **b** Quantification of collagen content in SD-sham (n = 5), SD-RDN (n = 5), ApoE^−\−^-sham (n = 5) and ApoE^−\−^-RDN (n = 5). Values are mean ± SEM. SD-sham vs. SD-RDN, p = 0.07 (n.s.); *p < 0.05; **p < 0.01

In 16 weeks old ApoE^−/−^ and SD rats, microscopic analysis of the aorta showed no detectable atherosclerotic plaque formation in the open luminal surface after staining with Oil-red O, and no visible fragmentation of elastin fibers after staining with Elastin van Gieson (Additional file 1: Figure S2). However, Sirius-red staining demonstrated an increased amount of interstitial fibrosis in the aortic media of ApoE^−/−^-sham compared with SD-sham (Fig. 5a). Collagen content was significantly lower in ApoE^−/−^-RDN compared with ApoE^−/−^-sham (Fig. 5b). Wall thickness of the aortic media, assessed by hematoxylin-eosin staining, was unchanged in ApoE^−/−^-sham compared to SD-sham (124 ± 9.8 vs. 105 ± 1.7 µm; p = 0.13), and was not influenced by RDN (SD-RDN: 103 ± 5.8 µm; ApoE^−/−^-RDN: 113 ± 7.3 µm; Additional file 1: Figure S2).

## Discussion

The sympathetic nervous system (SNS) is involved in the induction and progression of multiple cardiovascular pathologies such as vascular damage [7, 8], atrial fibrillation [14], increased heart rate [21], and hypertension [22]. Modulation of the SNS by RDN has been shown to reduce BP in certain patients with uncontrolled hypertension [9–12]. Recent studies have demonstrated that RDN may also exhibit additional systemic effects, occurring either in combination with blood-pressure lowering [13, 14], such as improvement of arterial stiffness in patients with hypertension [15], or being partly independent of blood pressure changes [16–18]. The underlying mechanisms of these beneficial effects on vascular compliance are currently incompletely understood. The present study therefore aimed to further elucidate on the role of the SNS and its modulation by RDN on aortic remodeling and compliance in a atherosclerosis prone animal model of hyperlipidemia. We used a novel normotensive, hypercholesterolemic ApoE-deficient rat model [19], and demonstrated that these rats exhibit severely decreased aortic distensibility and an impaired vasorelaxation, already at a stage before atherosclerotic lesions could be detected histologically in the aortic sinus or the thoracic aorta. Our main findings are, that sympathetic modulation by surgical bilateral RDN improved aortic compliance and attenuated endothelial dysfunction. These improvements were associated with a decrease in circulating aldosterone levels, reduced aortic medial fibrosis formation, as well as a lower inflammatory cytokine expression in circulating mononuclear cells (MNCs).

The renal SNS controls the renin secretion from the kidneys, thereby regulating the activity of the renin-angiotensin-aldosterone system (RAAS). Renin release stimulates the generation of angiotensin II (AngII), and the release of aldosterone from the adrenal cortex [23]. AngII has been linked to structural alterations of the aortic wall, characterized by elastin deposition, increase in medial wall thickness, and vascular fibrosis formation [24], accompanied by an enhanced expression of inflammatory cytokines, which in the long-term result in vascular stiffness [4, 6, 24, 25]. Accordingly, blockade of AngII decreases medial wall thickness and vascular fibrosis [20, 24, 25]. Selective aldosterone receptor blockade by eplerenone has been shown to reduce atherosclerosis and improve vasorelaxation in non-human primates fed with a high-cholesterol diet [26]. In ApoE^−/−^ mice, administration of eplerenone prevented atherosclerosis progression without affecting serum cholesterol or triglyceride levels [27]. This is in line with recent findings demonstrating that RDN decreases serum aldosterone and atherosclerotic lesion formation in the aortic roots of ApoE^−/−^-mice, independently of changes in BP [18]. Consistently with these reports, in the novel ApoE^−/−^ rat model used herein, RDN reduced plasma aldosterone levels and attenuated aortic medial fibrosis formation, while hyperlipidemia and cardiac parameters were not affected. Although RDN did not significantly influence blood pressure herein, it is conceivable that even a moderate decrease of BP may contribute to the herein observed improvements.

The aortic distensibility is codetermined by structural components of the arterial wall, i.e. extracellular matrix proteins [5, 28]. In the present study, ApoE^−/−^ rats demonstrated decreased aortic distensibility, associated with an increased aortic collagen content. This structural alteration was inhibited by RDN, preserving aortic distensibility, and emphasizes the influence of the SNS on aortic wall components. Vascular compliance, however, does not only depend on its wall composition, but also on endothelial function. Herein, modulation of the SNS by RDN positively influences endothelium-dependent relaxation which also might beneficially affect endothelial function. Endothelial function also highly depends on nitric oxide (NO)-bioavailability, regulated by the endothelial NO synthase (eNOS) [29, 30]. In ApoE-mice fed with high-fat diet down-regulation of eNOS was associated with impaired vascular function [20]. However, in our ApoE-deficient rat model, eNOS mRNA expression was not altered. It may also be conceivable, that local AngII mediates uncoupling of eNOS, thus decreasing total NO content and causing endothelial dysfunction [31]. The mechanisms regulation endothelial function are manifold and need to be further elucidated for this novel rat model.

Inflammation was also shown to initiate structural changes in the composition of the arterial wall, contributing to arterial stiffening [6, 32]. Several cytokines (e.g. IL1β, IL6, and TNFα) are known to stimulate the expression of ICAM-1 and VCAM-1, two important molecules involved in the development of atherosclerosis [5, 33]. In ApoE^−/−^ rats, impaired vascular compliance was associated with an increased inflammatory response, probably influencing aortic distensibility by inducing vascular remodeling processes. In the aorta of 16 weeks old preatherosclerotic ApoE^−/−^ rats, IL1β was significantly up-regulated compared to SD rats, but transcription levels of TNFα, and pro-atherosclerotic ICAM-1 and VCAM-1 were not significantly increased, underpinning the absence of atherosclerotic plaques in the aortic lumen. In isolated mononuclear cells (MNCs), mRNA expression of TNFα and IL6 was sensitive to RDN, and upregulation of these inflammatory marker genes could be inhibited. In contrast to MNCs, RDN did neither affect plasma levels of IL6 and IL1β nor gene expression of inflammatory cytokines in aortic tissue. Thus, in atherosclerosis prone ApoE^−/−^ rats, RDN failed to improve overall inflammatory activation, triggered by hypercholesterolemia [34], but influenced activation of MNCs, while circulating interleukin levels and local aortic inflammatory activation was not modulated.

## Conclusions

ApoE^−/−^ rats fed with standard chow at an age of 16 weeks develop impaired vascular compliance comparable to other ApoE-deficient animal models [2, 20], yet at an atherosclerosis prone stage. Sympathetic modulation by RDN prevented increasing aortic stiffening and positively effects endothelium-dependent relaxation in ApoE^−/−^, independently of alterations in BP and HR. These changes were associated with a reduction in aldosterone levels, and aortic fibrosis formation. Our data provide further evidence of the vital impact of the SNS in mediating vascular function and disease. The effects of SNS activation on vascular compliance are multifactorial. However, further experiments are needed to elucidate the involved pathways of the potential impact of RDN. At an age of 16 weeks hyperlipidemic ApoE^−/−^ rats revealed decreased aortic distensibility, and impaired vasorelaxation, when fed with normal chow, thus providing a useful tool to study aortic compliance and vascular response at an atherosclerosis prone stage. Future studies are necessary to assess whether catheter-based RDN also exhibits beneficial effects on vascular compliance in patients with and without hypertension.

## Clinical perspective

Several clinical trials have demonstrated that catheter based RDN reduces blood pressure in certain patients with resistant hypertension [9–12]. In contrast, the recently published SYMPLICITY 3 trial showed no significant difference in blood pressure between patients receiving sham treatment or RDN [35]. Reduction of afferent sympathetic nervous system activity and thereby central sympathetic activity may provide beneficial effects beyond lowering of blood pressure, including improvements in glucose tolerance [11], attenuation of the progression of atherosclerosis [18], reduction in left ventricular hypertrophy [36], and antiarrhythmic effects [17, 37]. Studies in a rat model of metabolic syndrome, bilateral RDN significantly reduced plasma renin activity, and prevented progression of kidney injury and cardiac remodeling [16]. Early trials in surgical sympathetectomy in severe hypertension documented reduced morbidity and mortality even in patients without blood pressure changes following the operation [38]. We provide preclinical evidence suggestive of beneficial vascular effects associated with RDN, not exclusively related to alterations in blood pressure.

## Limitations

At an age of 16 weeks hypercholesterolemic ApoE^−/−^ rats demonstrated elevated plasma aldosterone levels compared to normocholesterolemic SD. This is in line with observations, that aldosterone production is sensitive to elevated low-density lipoprotein concentrations [39]. Herein, RDN did not affect plasma aldosterone concentrations in SD, but prevented a progressive increase in ApoE^−/−^, preserving aldosterone levels comparable to SD. The underlying mechanism remains speculative. In our normotensive rat model, renal tissue concentrations of NE, renin, and AngII did not differ between SD and ApoE^−/−^, and RDN caused a similar decrease of these parameters in both SD and ApoE^−/−^ without significantly reducing blood pressure. This is in agreement with observations from Wang et al. [18], who documented significantly reduced concentrations in serum aldosterone, renal NE, and plasma renin in normotensive ApoE^−/−^ mice following RDN, however blood pressure and systemic concentrations of NE, renin, and AngII remained unaffected [18]. Whether the decrease in plasma aldosterone levels occurs indirectly due to systemic regulatory adaptive changes or via additional beneficial effects remains unclear and deserves further studies.

## Additional file

10.1186/s12967-016-0914-9 Complementary methods on aortic wall examination and oil-red O staining. **Figure S1**. Demonstrating the effect of 0.3 % cholesterol on liver fat-content and on plaque formation in the thoracic aorta and aortic sinus. **Figure S2**. Depicts the examination of atherosclerotic plaques, elastic laminae and aortic wall thickness using either oil-red O staining, Hematoxylin and Eosin Staining or Elastica Van Gieson staining. **Table S1**. Shows plasma concentration of inflammatory cytokines IL6 and IL1b and aortic gene expression of IL1b, TNFa, ICAM-1, VCAM-1 and eNOS.

## Abbreviations

AF: atrial fibrillation
ApoE^−/−^: apolipoprotein E-deficient
BP: blood pressure
ED: end diastole
ES: end systole
eNOS: endothelial nitric oxide synthase
GAPDH: glyceraldehyd-3-phosphate-dehydrogenase
GPT: glutamat-pyruvat-transaminase
GOT: glutamat-oxalacetat-transaminase
HDL: high-density lipoprotein
HPLC: high pressure liquid chromatography
HR: heart rate
ICAM-: intercellular adhesion molecule
IL1β: interleukin 1 beta
LDL: low-density lipoprotein
LVESV: left ventricular end-systolic volume
LVEDV: left ventricular end-diastolic volume
MNCs: mononuclear cells
MRI: magnetic resonance imaging
NE: norepinephrine
NO: nitric oxide
PP: pulse pressure
RAAS: renin-angiotensin-aldosterone-system
RDN: renal denervation
ROS: reactive oxygen species
SD: Sprague–Dawley
SNS: sympathetic nervous system
TNFα: tumor necrosis factor alpha
VCAM-1: vascular cell adhesion molecule-1
