# Supplementary material for: Modulation of the sympathetic nervous system by renal denervation prevents reduction of aortic distensibility in atherosclerosis prone ApoE-deficient rats
Source: J Transl Med. 2016 Jun 8;14:167. doi: 10.1186/s12967-016-0914-9 (PMC4898354; doi:10.1186/s12967-016-0914-9)
Supplement: Supplementary file 1 — 10.1186/s12967-016-0914-9 Complementary methods on aortic wall examination and oil-red O staining. Figure S1. Demonstrating the effect of 0.3 % cholesterol on liver fat-content and on plaque formation in the thoracic aorta and aortic sinus. Figure S2. Depicts the examination of atherosclerotic plaques, elastic laminae and aortic wall thickness using either oil-red O staining, Hematoxylin and Eosin Staining or Elastica Van Gieson staining. Table S1. Shows plasma concentration of inflammatory cytokines IL6 and IL1b and aortic gene expression of IL1b, TNFa, ICAM-1, VCAM-1 and eNOS. [file 12967_2016_914_MOESM1_ESM.docx]

**Online Data Supplements**

**Modulation of the sympathetic nervous system by renal denervation prevents reduction of aortic distensibility in atherosclerosis prone ApoE-deficient rats**

Mathias Hohl^1^, Dominik Linz^1^, Peter Fries^2^, Andreas Müller^2^, Jonas Stroeder^2^, Daniel Urban^1^, Thimoteus Speer^3^, Jürgen Geisel^4^, Björn Hummel^5^, Ulrich Laufs^1^, Stephan H. Schirmer^1^, Michael Böhm^1^, Felix Mahfoud^1^

^1^Klinik für Innere Medizin III, IMED, Universität des Saarlandes, Homburg/Saar, Germany

^2^Klinik für Diagnostische und Interventionelle Radiologie, Universitätsklinikum des Saarlandes, Homburg/Saar

^3^Klinik für Innere Medizin IV, Universitätsklinikum des Saarlandes, Homburg/Saar, Germany

^4^Zentrallabor, Klinische Chemie und Laboratorium Medizin, Universitätsklinikum des Saarlandes, Homburg/Saar, Germany

^5^Institut für Klinische Hämostaseologie und Transfusionsmedizin, Universitätsklinikum des Saarlandes, Homburg/Saar, Germany

Correspondence

Mathias Hohl, PhD

Klinik für Innere Medizin III, IMED

Universität des Saarlandes

66421 Homburg/Saar, Germany

phone: +49-6841-16-15155

fax: +49-6841-16-13211

Email: [Mathias.Hohl@uks.eu](mailto:Mathias.Hohl@uks.eu)

*Methods:*

*Aortic wall examination*

Thoracic aorta was sectioned (10 μm) on a Leica cryostat at −25°C (Kryostat Leica CM 1900-V5.0, Leica Microsystems, Nussloch, Germany). Aortic sections were stained with hematoxylin and eosin (H&E) to determine aortic wall thickness by computer-directed image analysis (NIS-Elements). The media aortic thickness was calculated as the mean of maximal and minimal thickness in aortic cross sections. Elastin van Gieson stain (Morphisto^®^, Frankfurt am Main, Germany) was used to show details of the elastic laminae.

*Oil-red O staining*

Thoracic aorta, aortic sinus, and liver tissue was treated as described above and stained with a 0.5% oil-red O working solution for 30 minutes. At least five consecutive sections per animal per staining were used for detection of atherosclerotic lesions in the thoracic aorta. Morphometric analysis was performed using the NIS-Elements analysis software.


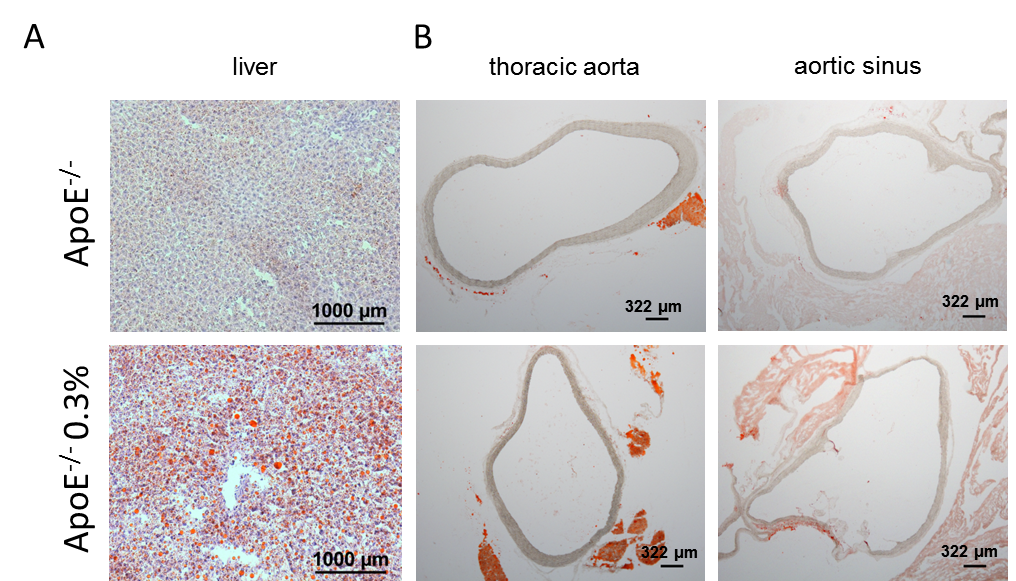


Figure S1:

Administration of Western-type diet containing 0.3% cholesterol to ApoE-deficient rats.

Oil red staining of (**A**) liver sections to visualize lipid-droplets and (**B**) of thoracic aortic and aortic sinus to detect atherosclerotic plaque formation.


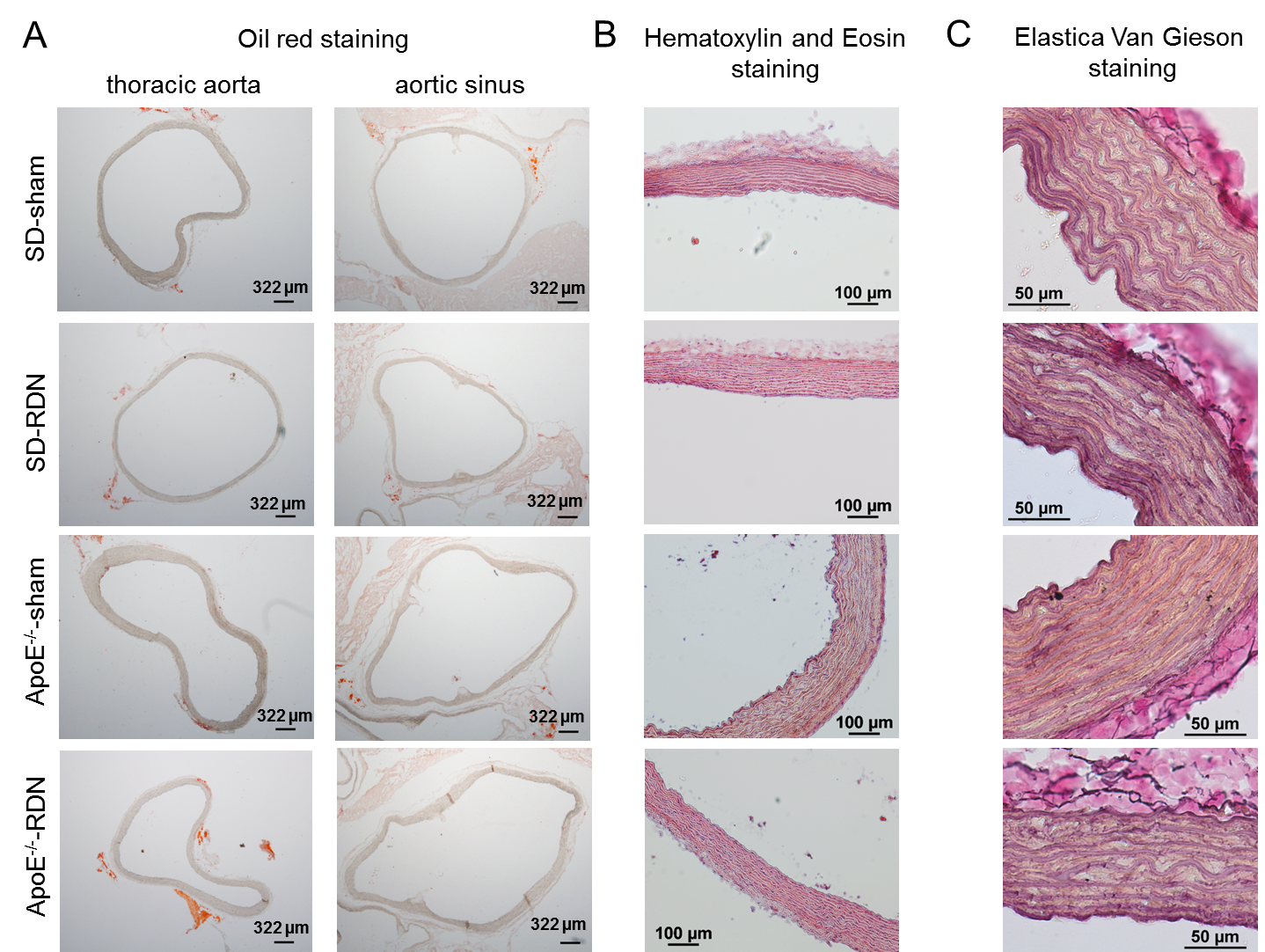


Figure S2:

Examination of atherosclerotic plaques, elastic laminae and aortic wall thickness. A) Determination of atherosclerotic lesions by Oil Red O-staining in thoracic aorta and aortic sinus. B) Hematoxylin-eosin staining, to assess wall thickness of the aortic media. C) Elastica van Gieson staining to analyze elastin fibers fragmentation in aortic sections from SD-sham, SD-RDN, ApoE^-\-^-sham and ApoE^-\-^-RDN.

Table S1: Plasma concentration and aortic gene expression of inflammatory markers

|  | **SD-sham** | **SD-RDN** | **ApoE^-/-^-sham** | **ApoE^-/-^-RDN** |
| --- | --- | --- | --- | --- |
| Plasma level |  |  |  |  |
| IL6 plasma [pg/ml] | 123.78±4.39 | 131.53±5.51 | 157.06±6.54** | 170.28±11.49 |
| IL1β plasma [pg/ml] | 65.75±15.32 | 62.79±9.76 | 177.41±8.77** | 172.64±26.57 |
| Aortic gene expression /GAPDH |  |  |  |  |
| IL1β | 1.15±0.29 | 1.14±0.33 | 2.22±0.37* | 2.06±0.34 |
| TNFα | 1.07±0.19 | 1.59±0.49 | 3.58±1.35 | 4.17±1.26 |
| ICAM-1 | 1.32±0.42 | 1.15±0.38 | 2.52±0.99 | 2.60±0.45 |
| VCAM-1 | 1.09±0.20 | 0.75±0.34 | 1.57±0.49 | 1.78±0.38 |
| eNOS | 1.11±0.28 | 0.97±0.30 | 0.99±0.30 | 1.48±0.23 |

IL6 (Interleukin 6); IL1β (Interleukin 1 beta), TNFα (Tumor necrosis factor alpha), ICAM-1 (intercellular adhesion molecule-1), VCAM-1 (vascular cell adhesion molecule-1), GAPDH (Glyceraldehyd-3-phosphate-dehydrogenase), eNOS (endothelial nitric oxidase synthase) *p<0.05, **p<0.01 SD-Sham vs. ApoE^-/-^ sham
